# Proteomic Identification of Nrf2-Mediated Phase II Enzymes Critical for Protection of Tao Hong Si Wu Decoction against Oxygen Glucose Deprivation Injury in PC12 Cells

**DOI:** 10.1155/2014/945814

**Published:** 2014-05-11

**Authors:** Hong-yi Qi, Li Li, Jie Yu, Lu Chen, Yong-liang Huang, Ling Ning, Zhuyun Jiang, Na Yang, Xiao-yu Xu

**Affiliations:** ^1^College of Pharmaceutical Sciences, Southwest University, 2 Tiansheng Road, Beibei District, Chongqing 400716, China; ^2^College of Pharmacy, Chengdu University of Traditional Chinese Medicine, Chengdu, Sichuan 611137, China; ^3^Institute of Laboratory Animals, Sichuan Academy of Medical Sciences & Sichuan Provincial People's Hospital, Chengdu, Sichuan 610212, China

## Abstract

Chinese herbal medicine formula Tao Hong Si Wu decoction (THSWD) is traditionally used in China for cerebrovascular diseases. However, the molecular mechanisms of THSWD associated with the cerebral ischemia reperfusion injury are largely unknown. The current study applied the two-dimensional gel electrophoresis-based proteomics to investigate the different protein profiles in PC12 cells with and without the treatment of THSWD. Twenty-six proteins affected by THSWD were identified by MALDI-TOF mass spectrometry. Gene ontology analysis showed that those proteins participated in several important biological processes and exhibited diverse molecular functions. In particular, six of them were found to be phase II antioxidant enzymes, which were regulated by NF-E2-related factor-2 (Nrf2). Quantitative PCR further confirmed a dose-dependent induction of the six phase II enzymes by THSWD at the transcription level. Moreover, the individual ingredients of THSWD were discovered to synergistically contribute to the induction of phase II enzymes. Importantly, THSWD's protection against oxygen-glucose deprivation-reperfusion (OGD-Rep) induced cell death was significantly attenuated by antioxidant response element (ARE) decoy oligonucleotides, suggesting the protection of THSWD may be likely regulated at least in part by Nrf2-mediated phase II enzymes. Thus, our data will help to elucidate the molecular mechanisms underlying the neuroprotective effect of THSWD.

## 1. Introduction

Traditional Chinese medicinal herbs have been commonly used in China and other Asian countries to combat various diseases for thousands of years. To make traditional Chinese medicine (TCM) become an evidence-based medicine, elucidation of the molecular mechanisms underlying the traditional effect of TCM with modern cutting-edge technologies has become a very urgent issue [1, 2]. Fortunately, the two-dimensional gel electrophoresis-based proteomic technique provides us with an approach for rapidly distinguishing altered cellular factors under drug treatment and thus opens up the possibility of understanding and interpreting TCM action mechanisms [3, 4].

Combination therapy with individual herbs to form specific formula has been used in TCM for about 2,500 years aiming to increase therapeutic efficacy and reduce adverse effects [5]. Tao Hong Si Wu decoction (THSWD) is a Chinese herbal medicine formula which is traditionally used for the prevention and treatment of cerebrovascular diseases in clinical practice in China. This formula contains six commonly used herbs, including Semen* Prunus* (Taoren in Chinese, TR), Flos* Carthami* (Honghua in Chinese, HH), Radix* Rehmanniae Preparata* (Shengdi in Chinese, SD), Radix* Angelicae Sinensis* (Danggui in Chinese, DG), Rhizoma* Ligustici Chuanxiong* (Chuanxiong in Chinese, CX), and Radix* Paeoniae Rubra* (Chishao in Chinese, CS) [6]. Our previous study showed that THSWD could regulate the angiogenesis, suggesting a potential application for the recovery after cerebral ischemia [7]. Recent research verified that THSWD exhibited neuroprotective activity against cerebral ischemia-reperfusion injury with the inhibition of HIF-1**α** and TNF-**α** activation as possible mechanisms [8].

To comprehensively elucidate the potential molecular mechanisms, the present study applied a proteomic strategy to well characterize the key molecular mechanisms of THSWD. First, we compared the proteomic profiles of PC12 cells treated with THSWD and vehicle, and then differentially expressed proteins were identified by MALDI-TOF mass spectrometry and annotated by gene ontology, and, finally, the key molecular mechanism was verified by confirmation of their expression level and biological role.

## 2. Materials and Methods

### 2.1. Chemicals and Antibodies

HO-1 antibody was purchased from Enzo Life Sciences (NY, USA). The antibodies against **β**-actin and rabbit IgG were purchased from Sigma-Aldrich (St. Louis, MO, USA). Other chemicals were obtained from Sigma-Aldrich Co. (St. Louis, MO, USA) unless indicated otherwise.

### 2.2. Preparation of Botanical Extracts

The raw materials of THSWD were purchased from Sichuan Neautus Traditional Chinese Medicine Co., Ltd., which is a GMP certified pharmaceutical producer of herbal slices in China. The powdered sample of THSWD (100 g) consisting of SD, CS, DG, CX, TR, and HH with the ratio of 12 : 9 : 9.6 : 9 : 6 was immersed in 0.8 L deionized water for 1 h and extracted twice for 1 h. The extract solution was filtered and evaporated with a rotary evaporator and then dried by lyophilization. Finally, the above herbal mixture yielded an amount of dry extract of 41.3 g. The stock solution of THSWD was sterilized by 0.22 *μ*m membrane filter before drug treatment to PC12 cells.

### 2.3. Cell Culture

Rat pheochromocytoma PC12 cells were obtained from the American Type Cell Culture Collection (Manassas, VA) and maintained in Dulbecco's modified Eagle's medium (DMEM) supplemented with 10% horse serum (Invitrogen, USA), 5% fetal bovine serum (FBS) (Invitrogen, USA), and 1% penicillin/streptomycin (Invitrogen, USA) on collagen I-coated dishes at 37°C in a humidified 5% CO_2_ atmosphere.

### 2.4. Measurement of Cell Viability

Cell viability was evaluated by a Cell Counting Kit-8 (CCK-8) assay (Dojindo Laboratories, Kumamoto, Japan), which is based on the conversion of a water-soluble tetrazolium salt, 2-(2-methoxy-4-nitrophenyl)-3-(4-nitrophenyl)-5-(2,4-disulfophenyl)-2H-tetrazolium, monosodium salt (WST-8), to a water-soluble formazan dye upon reduction by dehydrogenases in the presence of an electron carrier [9]. Briefly, at the end of drug treatment, cells were washed and CCK-8 solution (10 *μ*L) was added to each well, followed by incubation for 3 h at 37°C. The absorbance at 450 nm was determined by a microplate reader (Biotek, USA). Cell viability was expressed as a percentage of that of the control (untreated) cells.

### 2.5. Procedure of Oxygen Glucose Deprivation-Reperfusion (OGD-Rep)

The* in vitro* ischemia-reperfusion model was set up by OGD-Rep treatment of PC12 cells as described previously [10]. Briefly, PC12 cells were first incubated in glucose-free DMEM and subsequently transferred into a Tri-Gas incubator (Heal Force, HF100) with 1% O_2_, 94% N_2_, and 5% CO_2 _for 8 h at 37°C. Sham OGD cultures were maintained in a normal oxygenated DMEM. Following the OGD treatment, cells were returned to the normoxic incubator with normal culture medium and incubated for another 24 h.

### 2.6. Two-Dimensional (2D) Gel Electrophoresis

PC12 cells were treated with THSWD (1 mg·mL^−1^) or vehicle alone at 37°C for 24 h. At the end of the treatment, the cells were harvested and washed twice with ice-cold PBS. The cells were pelleted and lysed with FOCUS Mammalian protein extraction kit (Sangon, Shanghai, China). The cellular proteins were recovered following centrifugation at 20,000 ×g for 60 min at 4°C, and protein concentration was measured using the Bradford assay. Proteins were separated by 2D electrophoresis essentially according to the manufacturer's instructions (GE Healthcare, USA). For isoelectric focusing (IEF), 300 *μ*g of cellular proteins was mixed with a rehydration solution containing 2 M thiourea, 7 M urea, 2% CHAPS, 0.5% immobilized-pH-gradient (IPG) buffer (pH 3.0–10.0), and 10 mM DTT. The IEF was subsequently carried out in 24 cm Immobiline DryStrips (pH 3.0–10.0) using an ETTAN IPGphor 3 apparatus (GE Healthcare, USA). The focusing was achieved sequentially at 30 V for 12 h, 500 V for 1 h, 1,000 V for 1 h, 8,000 V for 8 h, and 500 V for 4 h. When the IEF was completed, the individual strips were equilibrated in 2D Equilibration Buffer (Sangon, Shanghai, China). The free thiol groups were inactivated with 2.5% iodoacetamide in the same buffer for another 20 min. After the IPG strips were mounted on the top of the gels, proteins were resolved in 12.5% SDS gels using the SE-600 electrophoresis unit (GE Healthcare, USA). SDS-PAGE was first run at 15 A/gel for 0.5 h and then 30 A/gel until the bromophenol blue front reached the bottom of the gel.

### 2.7. Protein Imaging and Analysis

The proteins resolved in the gel were visualized by a standard silver staining procedure described in the manufacturer's instructions (Invitrogen, USA). The stained gels were scanned using ImageScanner III LabScan 6.0 (GE Healthcare) and analyzed using ImageMaster Platinum software version 7.0 (GE Healthcare). Image analysis included spot detection, spot editing, background subtraction, and spot matching. The resulting data were exported to Microsoft Excel for comparison.

### 2.8. In-Gel Proteolytic Digestion and Peptide Identification by MALDI-TOF Mass Spectrometry

An automated spot picker was used to recover the spots of interest from the 2D gels to corresponding siliconized Eppendorf tubes. The in-gel proteolytic digestion was performed following a protocol from Shevchenko et al. [11]. For MALDI-TOF/TOF MS, the peptides were mixed with an equal volume of MALDI matrix (10 mg·mL^−1^ *α*-cyano-4-hydroxycinnamic acid (Sigma) saturated with 50% acetonitrile/0.1% formic acid) and spotted onto the MALDI sample plates. MS measurements were carried out on an ABI 4700 Proteomics Analyzer with delayed ion extraction (Applied Biosystems, Foster City, CA). The MS/MS setting was 2 kV positive mode (CID on) and 5 monoisotopic precursors selected (*S*/*N* > 200). The calibration was performed using the calibration mixture 1 of 4,700 Proteomics Analyzer calibration mixtures (Applied Biosystems, Foster City, CA). The spectra were calibrated externally using P14R and insulin chain B oxidized from bovine pancreas (Sigma). Autolytic peaks of trypsin served as internal standards for mass calibration. All PMFs obtained were used to search the NCBInr database using Mascot Daemon (Matrix Science, London, UK) as a client attached to the Mascot search protocol. The database searches had peptide mass tolerance set at approximately ±0.1 Da and one missed cleavage site. The protein spots were annotated by searching gene ontology (GO) (http://www.geneontology.org/).

### 2.9. Quantitative Real-Time PCR Detection

Total RNA was extracted from PC12 cells by using TriZol RNA extraction reagent (Invitrogen, CA, USA). Single-strand cDNA was synthesized using SuperScript III reverse transcription reagent (Invitrogen, CA, USA) according to the manufacturer's protocol. The specific primer sequences for each PCR reaction were listed in Table 1. The quantitative real-time PCR was performed on an ABI 7300 PCR System (Applied Biosystems, USA) using GoTaq qPCR Master Mix (Promega BioSciences, USA). Standard curves of the target genes were constructed with results of parallel PCR reactions performed on serial dilutions of a standard DNA. Fold change values were calculated by comparative Ct analysis after normalizing for the quantity of an endogenous reference gene **β**-actin mRNA in samples.

### 2.10. Western Blotting Analysis

Protein expression was analyzed by western blotting as previously described [10, 12]. Briefly, thirty micrograms of the cellular proteins was resolved by electrophoresis in 10% SDS-polyacrylamide gel and subsequently transferred to polyvinylidene difluoride (PVDF) membrane. Following 1 h incubation in a fresh TBS buffer containing 0.1% Tween-20 and 5% BSA, the blots were probed with specific antibodies including anti-HO-1 or anti-**β**-actin antibody. The bound primary antibodies were detected by horseradish peroxidase conjugated anti-rabbit IgG accordingly. The activity of peroxidase on the blot was visualized by enhanced chemiluminescence (ECL) detection reagents (GE Healthcare, Sweden).

### 2.11. Decoy Design and Treatment

ARE decoy oligonucleotide (ODN) was used in this study to inhibit Nrf2-driven genes according to previous study [13]. Upper-strand and reverse-complement phosphorothioated ODNs were commercially synthesized and purified by Sangon Biotech Inc. (Shanghai, China). Double-stranded decoy ODNs were prepared by annealing complimentary single strands in sterile saline. In addition to the ARE decoy ODNs, a scrambled decoy OND (*mut *ODN) was used as control for specificity. The following sequences were used in these studies: ARE, 5′-CTAATGGTGACAAAGCAACTTT-3′ and its compliment and ARE* mut*, 5′-CGACTGCCTTCAAAATAACTTT-3′ and its compliment. The underlining indicates the ARE core binding sequence. To increase the delivery of ODNs into the cell, lipofectamine 2000 (Invitrogen, USA) was used in the transfection treatment. The ARE decoy and ARE* mut* ODNs were added to the cells at 100 nM in the presence of lipofectamine 2000. After 24 h of incubation, THSWD extracts were added directly to the medium.

### 2.12. Statistical Analysis

All data were presented as mean ± SD for three independent experiments. Statistical analysis was performed by two-tail Student's *t*-test. A *P* value of less than 0.05 was considered to be statistically significant.

## 3. Results

### 3.1. THSWD Protected PC12 Cells against OGD-Rep Induced Injury

**Figure 1:**
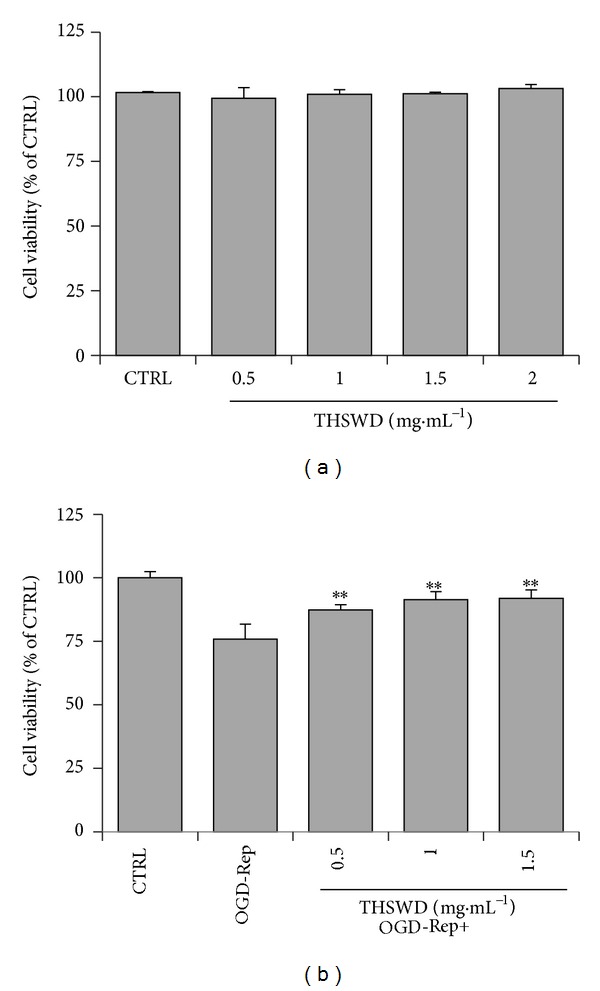
Cytotoxicity and protective effect of THSWD in PC12 cells. (a) Cytotoxicity of THSWD. PC12 cells were treated with THSWD at concentrations ranging from 0 to 2 mg·mL^−1^ for 24 h. (b) Effect of THSWD on the cell survival of PC12 cells against OGD-Rep. PC12 cells were pretreated with THSWD at concentrations ranging from 0 to 1.5 mg·mL^−1^ for 2 h, subsequently subjected to OGD-Rep treatment for 8 h, and finally maintained in oxygenated cell culture medium for another 18 h. The cell viability was determined by CCK-8 assay. Values represent mean ± SD (*n* = 6). **P < 0.01 versus OGD-Rep.

To examine the effect of THSWD on cellular viability, PC12 cells were treated with the drug for 24 h. The cell viability was determined using a CCK-8 assay. As shown in Figure 1(a), THSWD did not show toxicity up to the concentration of 2 mg·mL^−1^. We further addressed the question of whether THSWD could protect against ischemia-reperfusion-induced injury. We adopted an* in vitro* OGD-Rep procedure to mimic ischemic stroke as described [14]. As shown in Figure 1(b), OGD-Rep induced significant cell injury as indicated by CCK-8 assay. Notably, THSWD at concentrations ranging from 0.5 to 1.5 mg·mL^−1^ could significantly protect cells from OGD-Rep induced cell death (*P* < 0.01). Within the concentrations tested, THSWD showed a concentration-dependent response against OGD-induced injury in PC12 cells.

### 3.2. Proteomic Identification of the Proteins Mostly Affected by THSWD

**Figure 2:**
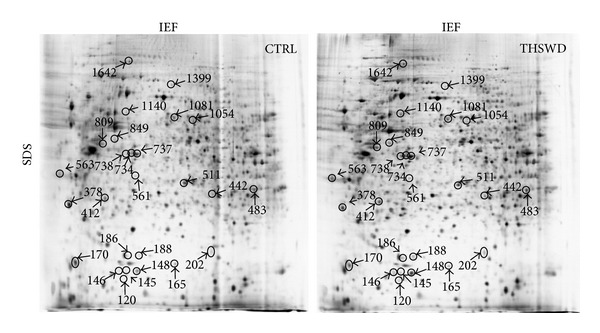
Representative proteome profiles of PC12 cells with and without exposure to THSWD. Cells were treated with THSWD (1 mg·mL^−1^) for 24 h and with vehicle as control. Total protein extracts were separated on pH 3–10 nonlinear IPG strips in the first dimension followed by 12.5% SDS-PAGE in the second dimension and were visualized by silver staining. A total of 26 differentially expressed spots were identified by MALDI-TOF/TOF mass spectrometer (marked with circle, arrow, and number).

To elucidate the molecular mechanisms underlying the protective effect of THSWD on OGD-Rep, we examined the effect of THSWD on the protein expression in PC12 cells. The cellular proteins obtained from THSWD-treated or untreated PC12 cells were resolved by 2D gel electrophoresis. Representative maps are shown in Figure 2. After spot detection, spot editing, and spot matching, the protein map of THSWD-treated cells was quantitatively compared with that of untreated cells using ImageMaster Platinum software version 7.0 (GE Healthcare). The average of spots resolved in these maps was about 2300. By applying a threshold of 1.2-fold variation, a total of 40 spots were identified as differentially expressed after THSWD treatment. Out of the 40 spots, 26 proteins were identified by MS analysis. There are 20 upregulated proteins and 6 downregulated proteins. The identified spots were marked with circles, arrows, and numbers (Figure 2), and the retrieved proteins corresponding to each numbered spot are listed in Table 2.

**Figure 3:**
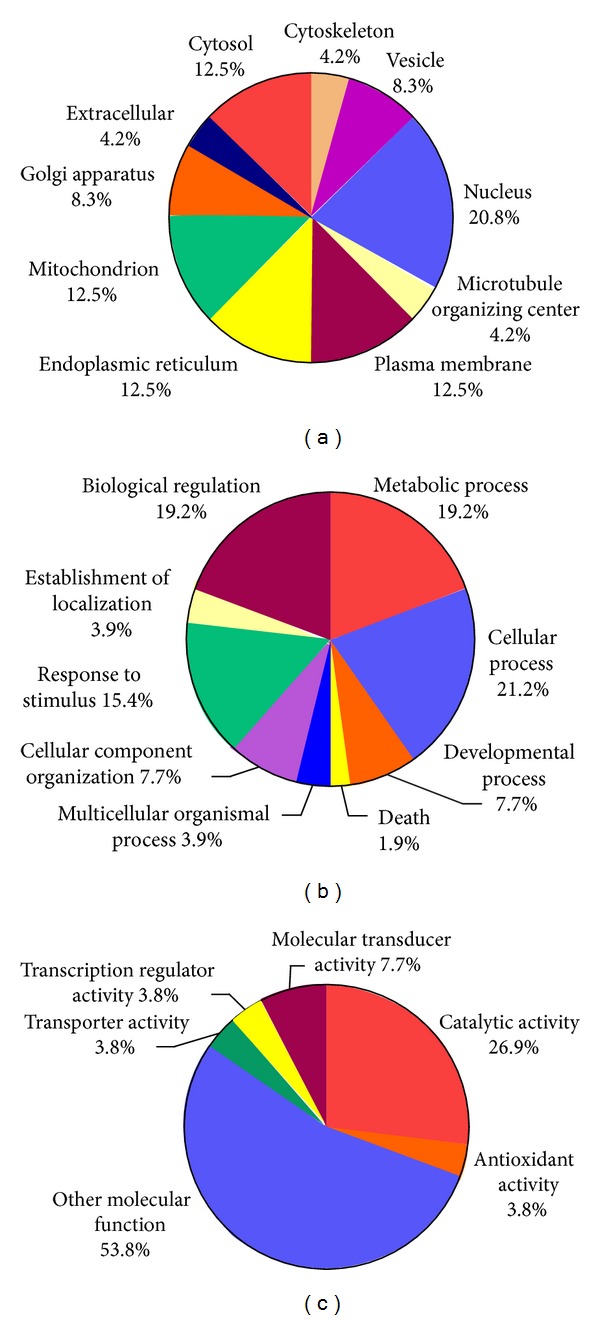
Gene ontology (GO) classification of the proteins affected by THSWD. (a) Cellular component; (b) biological process; and (c) molecular function. The percentage under the component, process, or function was the numerical proportion of proteins correlated to it to the total.

We further analyzed these proteins by the GO to classify their cellular components, biological processes, and molecular functions. As shown in Figure 3(a), a large percentage of identified proteins are located in a variety of cellular organelles (70.8%), including nucleus (20.8%), mitochondrion (12.5%), endoplasmic reticulum (12.5%), golgi apparatus (8.3%), vesicle (8.3%), cytoskeleton (4.2%), and microtubule organizing center (4.2%). The rest were from plasma membrane (12.5%), cytosol (12.5%), and extracellular region (4.2%). The biological processes of the identified proteins were diverse. However, a large percentage (76.9%) could be related to the cellular function during OGD-Rep induced injury: cellular process (in particular cellular homeostasis), metabolic process (in particular catabolic process and biosynthetic process), biological regulation (in particular regulation of metabolic process), response to stimulus (in particular response to stress), and death (Figure 3(b)). The identified proteins are found to be related to the varied molecular functions, including catalytic activity (26.9%), molecular transducer activity (7.7%), antioxidant activity (3.8%), transporter activity (3.8%), and transcription regulator activity (3.8%).

### 3.3. Discovery of Nrf2-Mediated Phase II Enzymes Induced by THSWD

**Figure 4:**
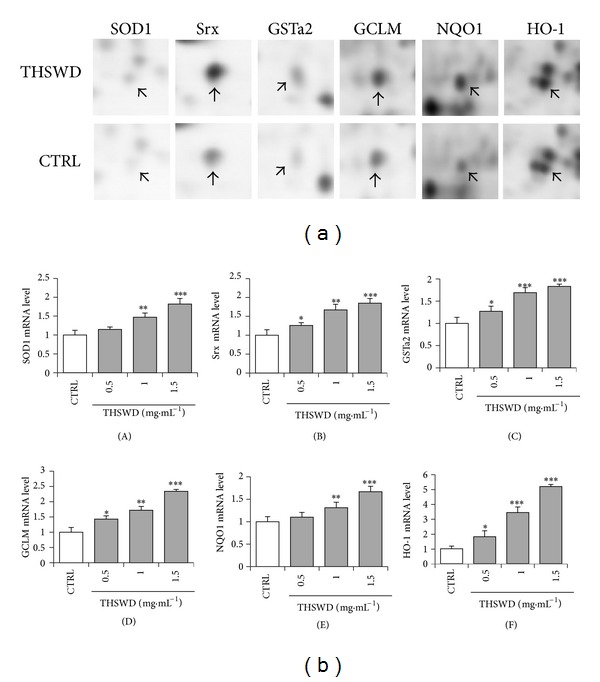
Discovery of Nrf2-mediated phase II enzymes induced by THSWD. (a) Enlargement of protein spots corresponding to Nrf2-driven proteins. The arrows indicate the target protein spots in THSWD-treated and control samples. (b) Quantitative real-time PCR analysis of the six genes corresponding to the 6 proteins directly regulated by Nrf2. After a 24 h induction by THSWD at indicated doses, SOD1 (A), Srx (B), GSTa2 (C), GCLM (D), NQO1 (E), and HO-1 (F) mRNA expression were determined by the quantitative real-time PCR system. Data represent the mean value of two biological replicates. Values represent mean ± SD (*n* = 3). *P < 0.05, **P < 0.01, and ***P < 0.001 versus vehicle control.

Among the 26 identified proteins, we found that 6 of them belong to transcription factor Nrf2-mediated phase II enzymes. These target proteins include superoxide dismutase [Cu–Zn] (SOD1), sulfiredoxin (Srx), glutathione S-transferase alpha-2 (GSTa2), glutamate-cysteine ligase regulatory subunit (GCLM), NAD(P)H dehydrogenase [quinone] 1 (NQO1), and heme oxygenase 1 (HO-1). The protein spots corresponding to these 6 proteins were carefully compared by amplifying the protein image (Figure 4(a)). To examine whether protein alterations observed by proteomic analysis correlate with the changes of those mRNAs at the transcription level, the 6 Nrf2-mediated proteins were further determined by quantitative real-time PCR. As shown in Figure 4(b), THSWD induced marked increased transcripts in a dose-dependent manner for all the six genes at concentrations ranging from 0.5 to 1.5 mg·mL^−1^. Our data demonstrated that THSWD induced alterations in the expression of those genes at both transcription and translational levels.

### 3.4. Synergistic Effect May Exist among the Individual Ingredients of THSWD on HO-1 Induction

**Figure 5:**
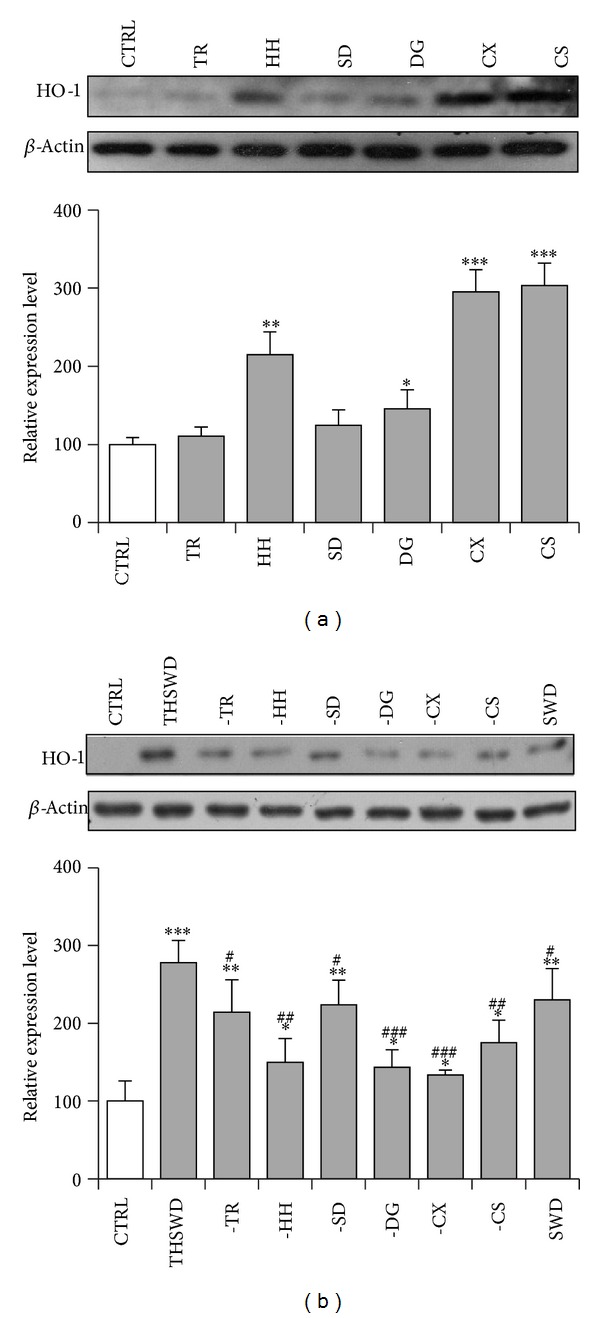
Contribution of individual ingredients in THSWD to the induction of HO-1 expression. (a) Determination of HO-1 inducing effect of individual ingredients. PC12 cells were treated with individual ingredients at equal concentration of 0.5 mg·mL^−1^ for 24 h. TR: Taoren (Semen* Prunus*); HH: Honghua (Flos* Carthami*); SD: Shengdi (Radix* Rehmanniae Preparata*); DG: Danggui (Radix* Angelicae Sinensis*); CX: Chuanxiong (Rhizoma* Ligustici Chuanxiong*); CS: Chishao (Radix* Paeoniae Rubra*). (b) Stepwise deletion of the ingredients from the parent THSWD formulation. PC12 cells were treated with new formulations deleting the indicated ingredients for 24 h. The concentration of each individual ingredient in new formulation is equal to that of parent formulation of 1 mg·mL^−1^. The cellular HO-1 protein was detected by western blotting using anti-HO-1 antibody, whereas**β**-actin was used as the control. “–”: minus the indicated ingredient from the whole formula. SWD: Si Wu decoction (a formula formed after deletion of TRand HH from THSWD). The blots were representative of three independent experiments. *P < 0.05, **P < 0.01, and ***P < 0.001 significantly different from vehicle group. ^#^ *P* < 0.05, ^##^ *P* < 0.01, and ^###^ *P* < 0.001 significantly different from THSWD group.

To determine the active ingredients in THSWD responsible for inducing Nrf2-mediated phase II enzymes, we first examined their inducing effect on HO-1, a key endogenous antioxidant and cytoprotective enzyme. PC12 cells were first treated with individual ingredients at equal concentrations (0.5 mg·mL^−1^). HO-1 protein expression was detected by western blotting using the specific antibody. Notably, CX and CS were found to be very strong inducers for HO-1 expression (*P* < 0.001). HH also significantly induced HO-1 expression but to a lesser extent (*P* < 0.01). DG showed the weak HO-1 inducing potency (*P* < 0.05), whereas the other ingredients showed no detectable induction of HO-1 expression (*P* > 0.05) (Figure 5(a)).

When used as a combination in 1 mg·mL^−1^of THSWD, the concentration of each individual ingredient is much less and is used according to the fixed ratio of the formula. We are curious about the contribution of each individual ingredient in THSWD to the HO-1 induction of the whole formula. Thus, a stepwise deletion strategy was applied. As presented in Figure 5(b), THSWD significantly induced HO-1 expression. Deletion of CX or DG caused very large decrease in the level of HO-1 protein (*P* < 0.001). Deletion of HH or CS also caused a large decrease but to a less extent (*P* < 0.01). When TR or SD, which showed no effect on HO-1 induction previously, was deleted, the capability of the parent formula in inducing HO-1 expression also reduced (*P* < 0.05). SWD, another formula used for improving women's health and formed after deletion of TR and HH from THSWD, showed less potency on HO-1 induction compared with that of THSWD (*P* < 0.05). Taken together, these results suggest that CX, DG, HH, and CS are the main active ingredients for inducing HO-1 expression in THSWD. However, the capability of the whole formula in inducing HO-1 expression seems to contain the contribution of all individual ingredients and synergistic effect may exist among them.

### 3.5. ARE Decoy Treatment Attenuated Protective Effect of THSWD against OGD-Rep Induced Injury in PC12 Cells

**Figure 6:**
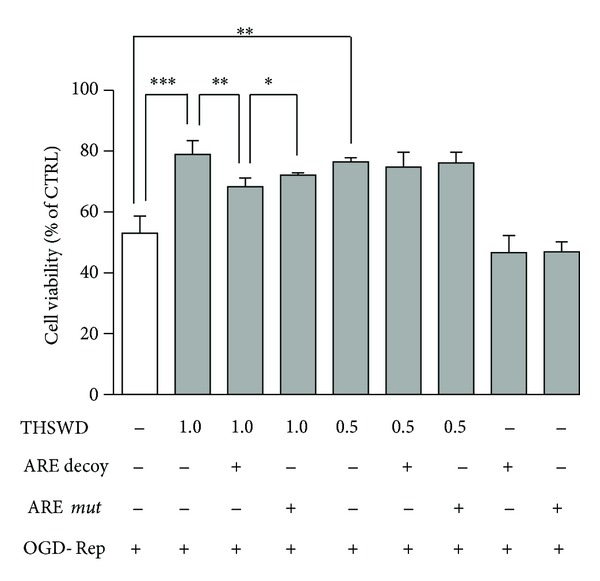
Role ofNrf2-mediated phase II enzymes in the protective effect of THSWD against OGD-Rep induced injury in PC12 cells. Cells were separately treated with two concentrations of THSWD (0.5 and 1.0 mg·mL^−1^) with and without ARE decoy ODNs or* mut* ODNs pretreatment. Subsequently, the cells were subjected to OGD for 8 h and finally maintained in oxygenated cell culture medium for another 24 h. The cell viability was determined by CCK-8 assay. Values represent mean ± SD (*n* = 6). *P < 0.05, **P < 0.01, and ***P < 0.001.

To further determine the role of Nrf2-mediated phase II enzymes in THSWD's protection against OGD-Rep induced injury, we tested the effect of THSWD in the presence and absence of ARE decoy ODNs, which could directly block the regulating effect of Nrf2 [13]. As shown in Figure 6, both 0.5 mg·mL^−1^ and 1.0 mg·mL^−1^ of THSWD significantly protected PC12 cells against OGD-Rep induced cell death. The protective effect of 1.0 mg·mL^−1^ THSWD significantly decreased in the presence of ARE decoy ODNs (*P* < 0.01), whereas the protective effect of 0.5 mg·mL^−1^ THSWD exhibited a decrease trend but without statistical significance in the presence of ARE decoy ODNs. Although ARE* mut* ODNs also reduced 1.0 mg·mL^−1^ THSWD mediated protection, the reducing extent caused by ARE decoy ODNs was significantly larger than that caused by ARE* mut* ODNs (*P* < 0.05). It is thus clear that ARE decoy ODNs significantly decreased THSWD's protection against OGD-Rep suggesting that this protection is likely mediated at least in part by Nrf2-driven phase II enzymes.

## 4. Discussion

A wide range of herbal medicines used in TCM and other folk medicines are found to be effective in the prevention and treatment of cerebral ischemia [15, 16]. Chinese herbal medicine formula THSWD is traditionally used in China for cerebrovascular diseases. Our previous study showed that THSWD could regulate the angiogenesis of chick chorioallantoic membrane, indicating the potential application for the recovery after cerebral ischemia [7]. Another previous study has verified that THSWD exhibited neuroprotective activity against cerebral ischemia-reperfusion-induced injury [8]. The present study also confirmed this by discovering THSWD mediated protection against OGD-Rep induced injury in PC12 cells. To find out the key molecular mechanisms of THSWD, a 2D electrophoresis-based proteomics approach was utilized to annotate the altered proteins in PC12 cells with and without treatment of THSWD. Twenty-six proteins affected by THSWD were identified by MALDI/MS technology.

The GO analysis showed that the identified proteins participated in several biological processes and exhibited diverse molecular functions. Regarding the cellular process (in particular cellular homeostasis), SOD1 binds copper and zinc ions and is one of three superoxide dismutases responsible for destroying free superoxide radicals and maintaining redox homeostasis in the body. Faster recovery of cerebral perfusion was observed in SOD1-overexpressed rats after cardiac arrest and resuscitation [17]. Brain infarction was significantly reduced in SOD1 transgenic mice after focal cerebral ischemia, while this effect disappeared in SOD1 transgenic mice after a permanent focal cerebral ischemia. The possible reason is that SOD1 mainly protects against reperfusion damage when oxidants are robustly generated, whereas permanent middle cerebral artery occlusion model does not involve reperfusion [18, 19]. Glial fibrillary acidic protein (GFAP) epsilon is a splice variant of GFAP, which is located in the central nervous system (CNS) and involved in many important CNS processes, including cell communication and the functioning of the blood brain barrier [20]. Regarding metabolic process (in particular catabolic process and biosynthetic process), HO-1 is an inducible enzyme that catalyzes the rate-limiting reaction in the metabolism of intracellular heme, leading to the generation of biliverdin, carbon monoxide, and free iron, which are potent antioxidant and cytoprotective molecules against oxidative injury [21, 22]. GCLM is the light regulatory subunit of glutamate-cysteine ligase, which catalyzes the rate-limiting reaction in glutathione (GSH) biosynthesis. It is observed that GCLM (−/−) mice were susceptible to ischemia-reperfusion injury, as an increased vulnerability of mitochondria to oxidative damage owing to mitochondrial GSH reduction [23]. Regarding biological regulation, transcription factor early growth response 3 (Egr3) has an essential role in VEGF-induced angiogenesis involved in vascular repair and neurovascular disease [24] and also regulates some aspects of synaptic plasticity related to learning and memory, in addition to controlling a wide variety of processes including biological rhythm, muscle development, and lymphocyte development [25]. Protooncogene c-Fos is critical in regulating the development of cells destined to form and maintain the skeleton and plays an important role in signal transduction and cell proliferation and differentiation [26]. Proliferation-associated protein 2G4 may play a role in an ERBB3-regulated signal transduction pathway and is involved in growth regulation [27, 28]. Regarding response to stimulus (in particular response to stress), heat shock protein beta-8, mitochondrial 60 kDa heat shock protein, and pre-mtHSP70 are a group of chaperonic proteins. Cerebral ischemia induces the toxic accumulation of unfolded proteins in the cytoplasm, mitochondria, and ER, which can trigger the induction of chaperonic proteins as prosurvival pathway [29]. Increased chaperonic activity makes cells better equipped to maintain the critical proteins in optimally folded state under stress [30]. Oxidative stress was one of the major factors causing cell death after cerebral ischemia [31]. Downregulation of most of the antioxidative proteins was found to be positively related to the severity of the induced oxidative stress [32, 33]. Antioxidant and detoxification phase II enzymes are known as the main cellular defense against oxidative stress [34]. Among our identified proteins, we surprisingly found that 6 of them (SOD1, Srx, GSTa2, GCLM, NQO1, and HO-1) belong to these phase II enzymes. Activation of phase II enzymes requires occupancy of a key regulatory element ARE in the promoter region by the basic region-leucine zipper transcription factor, Nrf2, which is known as the major mechanism of cellular defense against oxidative stress [35]. Therefore, we mainly focused on the Nrf2 pathway in the following investigation, but the other 20 proteins could not be excluded and will be taken into consideration in our future investigation.

To examine whether protein alterations observed by proteomic analysis correlate with the changes of those mRNAs at the transcription level, the 6 Nrf2-mediated proteins were further determined by quantitative real-time PCR. Our results demonstrated that THSWD enhanced the expression of these phase II enzymes in a dose-dependent way at mRNA level, whereas HO-1 showed the largest fold change upon the induction of THSWD. We then determined the contribution of each individual ingredient to the induction of phase II enzymes with HO-1 as representative. To our surprise, although only CX, DG, HH, and CS showed HO-1 inducing ability, it seems that all ingredients contributed to the HO-1 induction of the whole formula and a synergistic effect may exist among the individual ingredients. This is in accordance with previous reports that synergistic effect usually exists among the combination or multicomponents of herbal medicines [5, 36–38].

Pharmacological intervention activating Nrf2 signaling pathway is suggested as a promising approach for limiting oxidative stress after ischemia reperfusion [39]. Our proteomic analysis showed THSWD induced a group of Nrf2-mediated phase II enzymes. Therefore, we further determined the role of Nrf2 signaling pathway in the protective effect of THSWD against OGD-Rep induced injury. Thus, a transcription factor decoy approach was applied in our study. Sequence-specific inhibition of Nrf2 can be accomplished with synthetic double-stranded phosphothiorate oligonucleotides containing the ARE core binding sequence, which acts as a “decoy”* cis* element (ARE decoy ODNs) to bind transcription factor Nrf2 and blocks the activation of cognate genes [13, 40]. By using ARE decoy ODNs, we found that the protective effect of THSWD significantly decreased in the presence of ARE decoy ODNs. The* mut* ODNs also slightly reduced the effect of THSWD due to the cytotoxicity caused by transfection reagent. However, the significant difference between the reducing potency of decoy ONDs and* mut* ODNs could still reflect the role of Nrf2-mediated phase II enzymes induced by THSWD. These results demonstrated that Nrf2-mediated phase II enzymes may be in part responsible for the protective effect of THSWD against OGD-Rep induced injury and other protective mechanisms may also be involved. Although the neuronal-like PC12 cells are widely used as the cell model of neuroprotection, the primary neuronal cells and rodent brain ischemic model should be expected to be used for our further investigation of the protective effect of THSWD against cerebral ischemia. Based on the cell viability results in Figure 1(b), we used 1.0 mg·mL^−1^ THSWD, which showed no statistical difference in the protection from 1.5 mg·mL^−1^ THSWD, in the following proteomic study. However, the qPCR results showed that mRNA expression of some target genes could be induced significantly higher by 1.5 mg·mL^−1^ than by 1.0 mg·mL^−1^, which indicated that higher concentration (such as 1.5 mg·mL^−1^) of THSWD may result in stronger potency. Thus, higher concentration of THSWD should also be taken into consideration in our further study in cell model and animal model.

## 5. Conclusion

In conclusion, our proteomic analysis demonstrated that THSWD altered the expression levels of several biologically important proteins in PC12 cells. Especially, six phase II enzymes were discovered to be induced by THSWD at both transcription and translational levels. Moreover, the individual ingredients of THSWD were found to synergistically contribute to the induction of HO-1 by the whole formula. Finally, we verified that the protection of THSWD against the OGD-Rep induced injury may be likely regulated at least in part by Nrf2-mediated phase II enzymes. These results provide a better understanding of the molecular mechanisms underlying the traditional use of THSWD.

## Figures and Tables

**Table 1:** Primers used for quantitative real-time PCR.

Gene	Product length	Primer sequences	
SOD-1	129 bp	F	5′-CCACGAGAAACAAGATGACT-3′
		R	5′-GACTCAGACCACATAGGGAAT-3′

Srx	107 bp	F	5′-GACGTCCTCTGGATCAAAG-3′
		R	5′-GCAGGAATGGTCTCTCTCTG-3′

GSTa2	231 bp	F	5′-GGCAAAAGACAGGACCAAAA-3′
		R	5′-GGCTGCAGGAACTTCTTCAC-3′

GCLM	270 bp	F	5′-CTGACATTGAAGCCCAGGAG-3′
		R	5′-ACATTGCCAAACCACCACA-3′

NQO1	190 bp	F	5′-CGCAGAGAGGACATCATTCA-3′
		R	5′-CGCCAGAGATGACTCAACAG-3′

HO-1	107 bp	F	5′-ACCCCACCAAGTTCAAA CAG-3′
		R	5′-GAGCAGGAAGGCGGTCTTAG-3′

**β**-Actin	231 bp	F	5′-GGGGTGTTGAAGGTCTCAAA-3′
		R	5′-TGTCACCAACTGGGACGATA-3′

F: forward primer; R: reverse primer; SOD-1: superoxide dismutase [Cu–Zn]; Srx: sulfiredoxin; GSTa2: glutathione S-transferase alpha-2; GCLM: glutamate cysteine ligase, modifier subunit; NQO1: NAD(P)H quinone oxidoreductase 1; HO-1: heme oxygenase 1.

**Table 2:** MALDI/MS identification of the protein spots in response to THSWD.

Spot number	Fold change	Ther. *M*_*w*_/pI^a^	Protein description	Accession number	Pep. number^b^	Score
120	1.3	15912/5.88	Superoxide dismutase [Cu–Zn]	P07632	3	89
145	1.6	15145/5.65	Beta-actin FE-3	Q99NC6	1	50
146	1.7	21592/4.92	Heat shock protein beta-8	Q9EPX0	1	88
148	1.4	14194/6.30	Sulfiredoxin	B3DM86	3	102
165	−1.2	22254/9.19	Major prion protein	P13852	2	58
170	−1.6	21249/5.00	rCG50690	EDL86882	1	43
186	1.00*E* + 06	32711/4.62	Nucleophosmin	P13084	1	77
188	3	18561/6.27	Glial fibrillary acidic protein epsilon	A1E252	1	52
202	1.5	25428/8.88	Glutathione S-transferase alpha-2	P04903	2	75
378	−1.3	36412/8.33	Protein phosphatase 1 regulatory subunit 3C	Q5U2R5	3	87
412	1.3	30548/5.36	Glutamate-cysteine ligase regulatory subunit	P48508	2	70
442	1.8	74099/5.97	Pre-mtHSP70	P48721	1	102
483	1.2	30868/9.34	NAD(P)H dehydrogenase [quinone] 1	P05982	2	83
511	1.3	33006/6.09	Heme oxygenase 1	P06762	2	75
561	1.3	32622/6.42	Stanniocalcin-2	Q9R0K8		
563	1.3	40927/4.81	Protooncogene c-Fos	P12841	3	116
734	1.3	41643/5.31	Actin, gamma-enteric smooth muscle	P63269	2	95
737	1.3	42606/8.62	Early growth response protein 3	P43301	3	70
738	1.3	43657/6.41	Proliferation-associated 2G4	Q6AYD3	6	74
809	1.6	32894/4.86	Heterogeneous nuclear ribonucleoprotein C (C1/C2)	G3V9R8	3	136
849	1.00*E* + 06	38409/5.2	Suppressor of G2 allele of SKP1 homolog	B0BN85	2	53
1054	−2.4	50136 /4.9	Tubulin alpha-1A chain	P68370	2	100
1081	1.3	56573/8.6	G protein-activated inward rectifier potassium channel 1	P63251	4	167
1140	1.5	57926/5.35	60 kDa heat shock protein, mitochondrial	P63039	3	152
1399	−1.5	86990/7.62	Protein Igdcc3	D3ZQ86	4	129
1642	−1.3	108803/5.54	Ephrin type-A receptor 7	P54759	5	147

^a^Theoretical molecular weight (kDa) and pI from the ExPASy database.

^b^The number of unique peptides identified by MS/MS sequencing.
